# Association of maternal age with adverse pregnancy outcomes: A prospective multicenter cohort study in China

**DOI:** 10.7189/jogh.13.04161

**Published:** 2023-12-01

**Authors:** Yubo Zhou, Shaohua Yin, Qing Sheng, Jing Yang, Jianmeng Liu, Hongtian Li, Pengbo Yuan, Yangyu Zhao

**Affiliations:** 1 Institute of Reproductive and Child Health/National Health Commission Key Laboratory of Reproductive Health, Peking University Health Science Center, Beijing, China; 2 Department of Epidemiology and Biostatistics, School of Public Health, Peking University Health Science Center, Beijing, China; 3 Department of Obstetrics and Gynecology, Peking University Third Hospital, Beijing, China; 4 National Center for Healthcare Quality Management in Obstetrics, Peking University Third Hospital, Beijing, China; 5 National Clinical Research Centre for Obstetrics and Gynecology, Peking University Third Hospital, Beijing, China

## Abstract

**Background:**

Although maternal age might affect pregnancy outcomes, it remains unclear whether this relationship is linear or curvilinear and if it differs between nulliparous and multiparous women. We aimed to characterize the relationship between maternal age and risks of pregnancy outcomes in a diverse sample of Chinese singleton pregnant women and to evaluate whether the relationship varied by parity.

**Methods:**

We based this prospective multicenter cohort study on data from 18 495 singleton pregnant women who participated in the University Hospital Advanced Age Pregnant Cohort Study, conducted in eight Chinese public hospitals from 2016 to 2021. We used restricted cubic splines to model nonlinear relationships between maternal age continuum and adverse outcomes, and performed multivariable log-binomial regression to estimate the adjusted relative risk (RR) and 95% confidence interval (CI).

**Results:**

Among 18 495 singleton pregnant women (mean age 35.7, standard deviation (SD) = 4.2 years), maternal age was not related to postpartum hemorrhage or small for gestational age, but showed a positive, nonlinear relationship to gestational diabetes mellitus, hypertensive disorders of pregnancy, preeclampsia, placenta accreta spectrum, placenta previa, cesarean delivery, preterm birth, large for gestational age, macrosomia, and fetal congenital anomaly, with inflection points around 35.6-40.4 years. Compared to women younger than 35 years, older women had higher risks of adverse pregnancy outcomes, except for postpartum hemorrhage and small for gestational age. The risks of placenta accreta spectrum, placenta previa, large for gestational age, and macrosomia were highest for women aged 40-44 years, and risks of gestational diabetes mellitus, hypertensive disorders of pregnancy, preeclampsia, cesarean delivery, preterm birth and congenital anomaly were highest for those aged ≥45 years. Most risks were more pronounced in nulliparous than multiparous women (*P* for interaction <0.02).

**Conclusions:**

Delayed childbirth was related to increased risks of adverse pregnancy outcomes, especially for nulliparous women. Appropriate childbearing age, generally before 35 years, is recommended for optimising pregnancy outcomes.

Adverse pregnancy outcomes remain a major global health threat [1,2]. They occur due to several factors, with maternal age being the most relevant one [3-5]; however, evidence on its association with pregnancy outcome is currently unsatisfactory for clinical consultation [3,6,7].

Previous studies used 35 years as the threshold of advanced maternal age, despite a lack of evidence on its biological plausibility [8]. Moreover, maternal age has usually been treated as a categorical variable, often divided into five-year increments, with few studies exploring its relationship as a continuous variable with pregnancy risks, which might differ by adverse outcomes. Furthermore, most research focused on Western populations, with few studies conducted in China [9], even though the associations, mediated by sociodemographic characteristics and clinical therapy, might differ between countries [3-5]. It is also unclear whether the associations vary by different maternal characteristics such as parity and pre-pregnancy body mass index (BMI).

In China, the recent fertility policy transition has led to changes in many maternal and pregnancy characteristics [10,11], with the most drastic one being the increasing maternal age. For example, the proportion of women who delivered at ≥35 years increased from 8.5% in 2013 to 15.8% in 2017 in Zhejiang province [12], and from 4.3% to 13.9% in the same period in Wuhan city [13]. As this transition period continues, it is crucial to comprehensively understand the relationships between maternal age and adverse pregnancy outcomes in Chinese women.

Using data from a prospective multicenter cohort in Chinese pregnant women, we aimed to quantify this linear or curvilinear relationships and identify the inflection point when the latter was observed. We also sought to explore whether the relationships were modified by maternal key characteristics such as parity, pre-pregnancy BMI, and pre-existing comorbidities.

## METHODS

### Study design and participants

We conducted a prospective multicenter study based on the University Hospital Advanced Age Pregnant (UNIHOPE) cohort conducted in China from July 2016 to June 2021 (ClinicalTrails No.: NCT03220750) [14]. Women in early pregnancy were recruited from eight large hospitals in seven cities, geographically distributed in eastern, central, and western China (Table S1 in the **Online Supplementary Document**). The cohort was designed to identify predictors of pregnancy complications and adverse outcomes among women of advanced age, given their increasing number following the implementation of the universal two-child policy in 2016. Therefore, UNIHOPE primarily included women in early pregnancy, receiving prenatal health care at the study hospitals, and with anticipated delivery date at age ≥35 years, but also a small proportion of younger women (<35 years at delivery) as potential comparisons. The study excluded women with mental disorders or those unable to provide informed consent.

After enrolment, pregnant women were followed up at 24-28 weeks, 32-34 weeks of gestation, delivery, and 6-12 weeks postpartum. Maternal sociodemographic characteristics (age, ethnicity, education, occupation, and annual household income), lifestyle (alcohol consumption and smoking within six months prior to pregnancy), medical and reproductive history (comorbidities before pregnancy, parity, and current method of conception), prenatal care of the current pregnancy (pre-pregnancy BMI, gestational age at enrolment), and pregnancy complications and delivery outcomes was collected using a structured questionnaire during the follow-ups by nurses or obstetricians.

**Figure 1:**
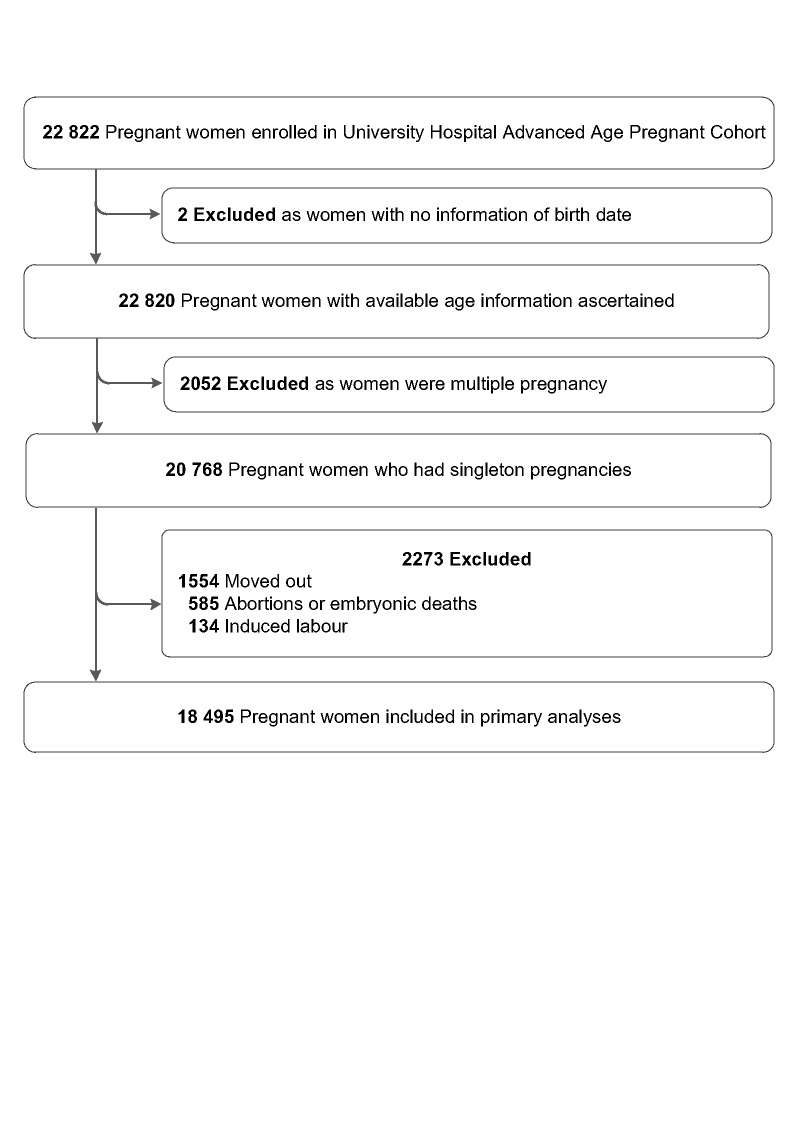
Flowchart of participants selection.

The UNIHOPE cohort initially enrolled 22 822 pregnant women. We excluded those with unavailable birth date (n = 2), with multiple pregnancy (n = 2052), with abortion or embryonic death (n = 585), with induced labor (n = 134), or moved out (n = 1554), leaving 18 495 women aged 19 to 54 years in the final analysis (**Figure 1**).

### Exposure and outcomes

We defined maternal age as the age of the mother at the time of delivery, calculated as subtracting the maternal birthdate from the expected date of delivery. We used it both as a continuous variable and a categorical variable for comparison with previous studies (19.0-34.9, 35-39.9, 40-44.9, and ≥45.0 years) [6,15].

Our outcomes of interest were gestational diabetes mellitus (GDM), preeclampsia (PE), hypertensive disorders of pregnancy (HDP), placenta accreta spectrum (PAS) disorders, placenta previa (PP), cesarean delivery, postpartum hemorrhage (PPH), preterm birth (PTB), large for gestational age (LGA), small for gestational age (SGA), macrosomia, and congenital anomaly.

GDM was defined as a glucose intolerance (a fasting plasma glucose ≥5.1 mmol/L or an oral glucose tolerance test (OGTT) one hour plasma glucose ≥10.0 mmol/L, or an OGTT two hour plasma glucose ≥8.5 mmol/L) that begins or is first recognized during pregnancy [16]. We defined PE as elevated blood pressure and proteinuria after 20 weeks of gestation [17] and HDP a group of high blood pressure disorders encompassing PE, superimposed on chronic hypertension, gestational hypertension, and chronic hypertension. PAS was a condition of abnormal placental invasion encompassing placenta accreta, increta, and percreta [18]. PP was diagnosed by clinical signs of echogenic homogeneous placental tissue extending over the internal cervical or on a second- or third-trimester imaging using transvaginal ultrasound. Cesarean delivery was identified by inpatient medical records. PPH was defined as an estimated blood loss of ≥500 ml following vaginal delivery or ≥1000 ml following cesarean delivery [19], measured with a graduated collector bag. PTB was defined as delivery less than 37 completed gestational weeks. LGA was defined as birthweight greater than the 90th percentile for gestational age, and SGA as birthweight less than the 10th percentile for gestational age, according to gender-specific Chinese curves [20]. Macrosomia was defined as birthweight ≥4000 g. Congenital anomaly was defined as structural anomalies identifiable for fetus or infants, such as congenital heart defect, Down syndrome, polydactylism, and cleft palate.

### Statistical analysis

We descriptively analyed maternal characteristics and pregnancy outcomes, presenting them as means with standard deviations (SDs) or medians with interquartile ranges (IQRs), depending on normality of distribution, as assessed by the Shapiro-Wilk test. We otherwise presented categorical variables as frequencies with percentages. We compared maternal age groups using the χ^2^ test and one-way analysis of variance or Kruskal-Wallis tests, as appropriate.

We used multivariable logistic regression models with restricted cubic spline approach to explore the nonlinear relationships between maternal age and adverse pregnancy outcomes. We determined the number of knots (between three and seven) based on the lowest Akaike information criterion [21] (Table S2 in the **Online Supplementary Document**) and applied the Markov Chain Monte Carlo (MCMC) algorithm to identify the inflection point, if any, when nonlinear association was observed. We calculated adjusted odds of each outcome in one-year increments of maternal age from 19 to 54 years and transformed them to predicted probabilities. The predicted probabilities with 95% confidence intervals (CIs) were plotted for maternal age to display the relationships visually. We also used univariable and multivariable log-binomial regression models to calculate relative risks (RRs) with 95% CIs for the associations between categorical maternal age and adverse pregnancy outcomes.

We evaluated whether the relationships or associations were modified by parity (nulliparous and multiparous women), pre-pregnancy BMI (underweight (<18.5 kg/m^2^), normal weight (18.5-24.9 kg/m^2^), overweight (25-29.9 kg/m^2^), and obesity (≥30 kg/m^2^) [22], or pre-existing comorbidities (no and yes) by adding an interaction term into the multivariable regression models. If a significant interaction was observed, subgroup analyses for the corresponding modifier was performed. To assess whether the relationships were influenced by coronavirus 2019 (COVID-19) pandemic, subgroup analyses stratified by COVID-19 (before and during COVID-19) were performed. Before COVID-19 was defined as from July 2016 to December 2019, and during COVID-19 as from January 2020 to June 2021.

All multivariable regression models were adjusted for ethnicity, education, occupation, annual household income, pre-pregnancy BMI, smoking or alcohol consumption, parity, method of conception, and year of delivery. We classified missing values of each covariate into a new category, when regression models applied [23] and performed further sensitivity analysis with the missing values imputed using multiple imputation [24].

We conducted all analyses in SAS software, version 9.4 (SAS Institute, Inc, Cary, North Carolina, USA). All tests were two-sided, and *P* < 0.05 was considered statistically significant.

## RESULTS

The 18 495 pregnant women had a mean age of 35.7 years (SD = 4.2, range = 19-54) years, with 25.1% (n = 4650), 59.5% (n = 11 008), 14.4% (n = 2670), and 0.9% (n = 167) being 19-34.9, 35-39.9, 40-44.9, and ≥45 years old, respectively. Most were of Han ethnicity (92.5%), had high school or above education (76.3%), middle or above annual household income (77.1%), normal pre-pregnancy weight (68.2%), were non-smoker (89.7%) and non-alcohol drinker (85.6%), multiparous (80.5%), and conceived spontaneously (78.4%). The maternal sociodemographic and clinical characteristics significantly varied across categories of maternal age (**Table 1**). High educational status, high annual household income, overweight/obesity prior pregnancy, morbidity prior pregnancy, multiparous women, and conceived using assisted reproductive technology were more common among women aged 35 years or older.

**Table 1:** Characteristics of the study participants*

	Maternal age, number (%)					
**General characteristics**	**Total (N = 18 495)**	**<35 (n = 4650)**	**35-39 (n = 11 008)**	**40-44 (n = 2670)**	**≥45 (n = 167)**	***P-*value**
Maternal age, year, mean (SD)	35.7 (4.2)	29.9 (2.8)	36.7 (1.3)	41.2 (1.2)	46.2 (1.8)	
Ethnicity						<0.001
*Han*	17 106 (92.5)	4099 (88.2)	10 369 (94.2)	2485 (93.1)	153 (91.6)	
*Other*	808 (4.4)	316 (6.8)	389 (3.5)	95 (3.6)	8 (4.8)	
*Missing*	581 (3.1)	235 (5.1)	250 (2.3)	90 (3.4)	6 (3.6)	
Education						<0.001
*Primary or less*	140 (0.7)	16 (0.3)	84 (0.8)	30 (1.1)	10 (6.0)	
*Secondary*	279 (1.3)	163 (3.5)	519 (4.7)	177 (6.6)	20 (12.0)	
*High school or above*	15 944 (76.3)	3862 (83.1)	9704 (88.2)	2254 (84.4)	124 (74.3)	
*Missing*	4532 (21.7)	609 (13.1)	701 (6.4)	209 (7.8)	13 (7.8)	
Occupation						<0.001
*Office worker*	8158 (44.1)	1605 (34.5)	5249 (47.7)	1236 (46.3)	68 (40.7)	
*Labor worker*	2654 (14.3)	733 (15.8)	1587 (14.4)	311 (11.6)	23 (13.8)	
*Others*	7010 (37.9)	2039 (43.8)	3881 (35.3)	1020 (38.2)	70 (41.9)	
*Missing*	673 (3.6)	273 (5.9)	291 (2.6)	103 (3.9)	6 (3.6)	
Annual household income						<0.001
*Low*	1868 (10.1)	510 (11)	1092 (9.9)	252 (9.4)	14 (8.4)	
*Middle*	7666 (41.4)	2296 (49.4)	4210 (38.2)	1073 (40.2)	87 (52.1)	
*High*	6611 (35.7)	976 (21.0)	4561 (41.4)	1025 (38.4)	49 (29.3)	
*Missing*	2350 (12.7)	868 (18.7)	1145 (10.4)	320 (12.0)	17 (10.2)	
Pre-pregnancy body mass index in kg/m^2^, mean (SD)						<0.001
*Underweight*	1418 (7.7)	514 (11.1)	765 (6.9)	132 (4.9)	7 (4.2)	
*Normal weight*	12 614 (68.2)	2871 (61.7)	7774 (70.6)	1859 (69.6)	110 (65.9)	
*Overweight*	2392 (12.9)	493 (10.6)	1465 (13.3)	403 (15.1)	31 (18.6)	
*Obesity*	431 (2.3)	122 (2.6)	243 (2.2)	64 (2.4)	2 (1.2)	
*Missing*	1640 (8.9)	650 (14.0)	761 (6.9)	212 (7.9)	17 (10.2)	
Smoking within six months before pregnancy						<0.001
*Non-smoker*	16 587 (89.7)	3912 (84.1)	10 114 (91.9)	2410 (90.3)	151 (90.4)	
*Smoker*	275 (1.5)	97 (2.1)	143 (1.3)	32 (1.2)	3 (1.8)	
*Missing*	1633 (8.8)	641 (13.8)	751 (6.8)	228 (8.5)	13 (7.8)	
Alcohol consumption within six months prior to pregnancy						<0.001
*Non–drinker*	15 832 (85.6)	3865 (83.1)	9532 (86.6)	2289 (85.7)	146 (87.4)	
*Drinker*	991 (5.4)	134 (2.9)	704 (6.4)	147 (5.5)	6 (3.6)	
*Missing*	1672 (9.0)	651 (14.0)	772 (7.0)	234 (8.8)	15 (9.0)	
Preexisting comorbidities†	3924 (21.2)	723 (15.6)	2543 (23.1)	620 (23.2)	38 (22.8)	<0.001
Gestational age at enrolment, mean (SD)	12.7 (4.7)	12.3 (5.0)	12.9 (4.6)	12.9 (4.7)	12.8 (4.6)	<0.001
Current pregnancy						
Parity						<0.001
*Nullipara*	3611 (19.5)	2071 (44.5)	1316 (12.0)	213 (8.0)	11 (6.6)	
*Multipara*	14 884 (80.5)	2579 (55.5)	9692 (88.0)	2457 (92.0)	156 (93.4)	
Method of conception						
*Spontaneous*	14 493 (78.4)	3657 (78.6)	8695 (79)	2043 (76.5)	98 (58.7)	
*Assisted reproductive technology*	2391 (12.9)	299 (6.4)	1615 (14.7)	419 (15.6)	58 (34.7)	
*Missing*	1611 (8.7)	694 (14.9)	698 (6.3)	208 (7.8)	11 (6.6)	
Delivery year						<0.001
*2017*	2488 (13.5)	528 (11.4)	1606 (14.6)	345 (12.9)	9 (5.4)	
*2018*	7786 (42.1)	1784 (38.4)	4712 (42.8)	1206 (45.2)	84 (50.3)	
*2019*	5016 (27.1)	600 (12.9)	3492 (31.7)	856 (32.1)	68 (40.7)	
*2020*	2047 (11.1)	1089 (23.4)	788 (7.2)	168 (6.3)	2 (1.2)	
*2021*	1158 (6.3)	649 (14.0)	410 (3.7)	95 (3.6)	4 (2.4)	

SD – standard deviation

*Data are presented as number (percentage) of deliveries unless otherwise indicated. Percentages have been rounded and may not total 100.

†Preexisting comorbidities was defined as the presence of the one of the following eight diseases recorded before pregnancy, including heart disease, renal disease, hepatic disease, immune system disease, reproductive system disease, thyroid disease, hypertension, and diabetes.

For most outcomes except for SGA, the prevalence of adverse pregnancy outcomes increased with maternal age, was highest in the 40-44.9- or ≥45-year-old groups (**Table 2**). For example, the prevalence of GDM was 18.3%, 27.8%, 33.9%, and 44.3% for women aged 19-34.9, 35-39.9, 40-44.9, and ≥45 years old, respectively.

**Table 2:** Maternal and neonatal outcomes across maternal age*

	Maternal age, number (%)					
	**Total (N = 18 495)**	**<35 (n = 4650)**	**35-39 (n = 11 008)**	**40-44 (n = 2670)**	**≥45 (n = 167)**	***P*-values for trend**
Gestational diabetes mellitus	26.4 (4891)	18.3 (849)	27.8 (3062)	33.9 (906)	44.3 (74)	<0.001
Hypertensive disorders of pregnancy	9.9 (1839)	5.9 (275)	10.7 (1180)	12.7 (340)	26.4 (44)	<0.001
Preeclampsia	4.6 (844)	2.8 (129)	4.9 (534)	6.0 (160)	12.6 (21)	<0.001
Placenta accreta spectrum	7.7 (1432)	3.0 (137)	8.8 (969)	11.2 (300)	15.6 (26)	<0.001
Placenta previa	7.7 (1432)	3.6 (168)	8.7 (957)	10.7 (285)	13.2 (22)	<0.001
Cesarean delivery	58.9 (10 894)	41.5 (1929)	62.1 (6840)	74.3 (1984)	84.4 (141)	<0.001
Postpartum hemorrhage	8.0 (1483)	5.8 (268)	8.6 (945)	9.4 (251)	11.4 (19)	<0.001
Preterm birth	11.2 (2072)	7.6 (353)	11.5 (1269)	15.5 (414)	21.6 (36)	<0.001
Large for gestational age†	19.2 (2833)	16.7 (527)	19.2 (1765)	22.9 (510)	21.1 (31)	<0.001
Small for gestational age†	6.6 (969)	6.4 (202)	6.8 (630)	5.6 (124)	8.8 (13)	0.613
Macrosomia	16.3 (3017)	12.5 (581)	17.0 (1867)	20.1 (537)	19.2 (32)	<0.001
Congenital anomaly	3.3 (607)	1.5 (70)	3.8 (421)	3.8 (102)	8.4 (14)	<0.001

*Data are presented as percentage (number) of deliveries unless otherwise indicated.

†Among 18 495 singleton women analyzed in this study, there were 3750 women (20.3%) with missing information on birthweight, neonatal sex, or gestational age at delivery who were not included when performing the description and comparison of small for gestational age and large for gestational age.

**Figure 2:**
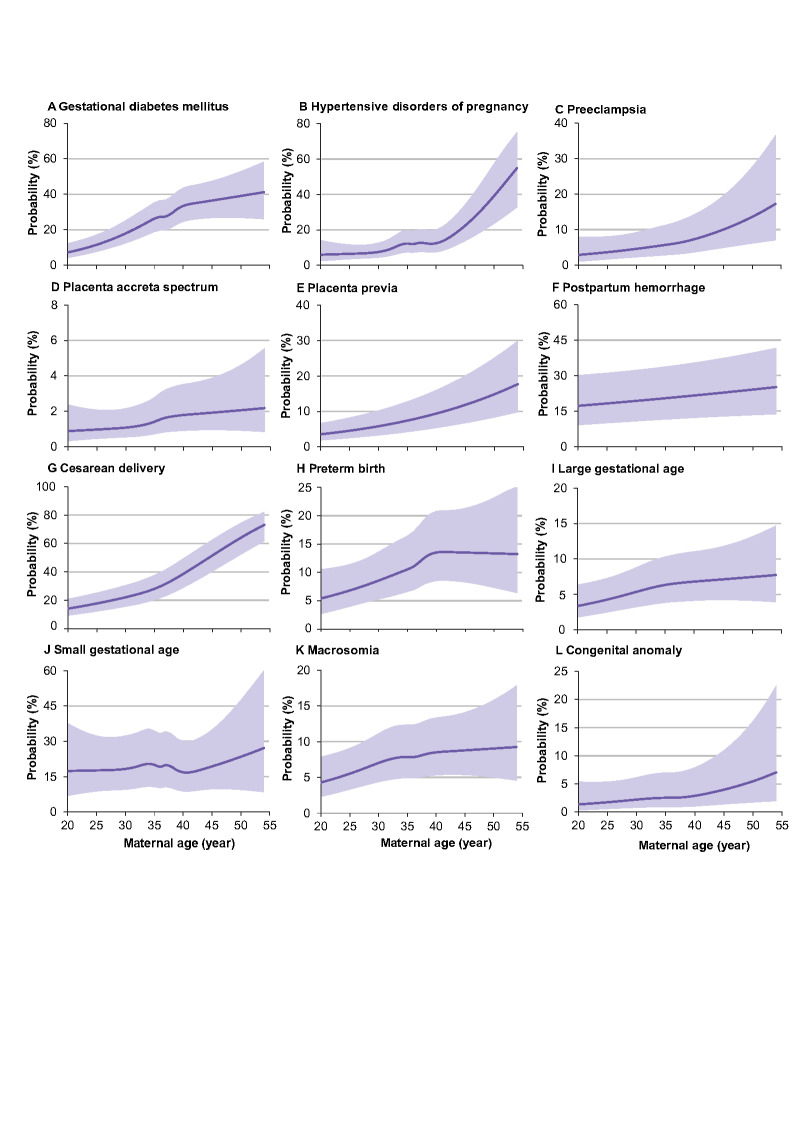
Predicted probabilities of adverse pregnancy outcomes across maternal age. Predicted probabilities were transformed from the adjusted odds, which were calculated using multivariable logistic regression models, with adjustment for ethnicity, education, occupation, annual household income, gestational age at enrolment, pre-pregnancy body mass index, smoking and alcohol consumption within six months prior to pregnancy, parity, method of conception, and preexisting comorbidities. Dark purple lines indicate predicted probabilities, and the purple bands represent 95% confidence intervals.

Analyses of maternal age as a continuous variable showed a nonlinear relationships with adverse pregnancy outcomes (**Figure 2**). Though the shapes of curves differed, the predicted probabilities increased with increasing maternal age for all outcomes except for PPH and SGA. The probability of GDM increased rapidly with maternal age up to a threshold of 40.4 years and then decelerated. The probabilities for LGA and PAS increased up to 35.6 and 36.4 years, and up to 40.0 to 40.3 years for macrosomia and PTB, and then plateaued. The probabilities for cesarean delivery and PP increased gradually with maternal age up to 36.2 and 36.4 years, and up to 39.6 to 40.4 years for HDP, PE, and congenital anomaly, and then accelerated. The risks related to increasing maternal age for GDM (*P* for interaction = 0.032), HDP (*P* for interaction = 0.002), PE (*P* for interaction = 0.018) and cesarean delivery (*P* for interaction <0.001) were higher in nulliparous women than multiparous women (Figure S1 in the **Online Supplementary Document****)**, and the risks for GDM (*P* for interaction <0.001) and PAS (*P* for interaction <0.001) were higher during than before COVID-19 (Figure S2 in the **Online Supplementary Document****)**. We observed no significant difference in risks across pre-pregnancy BMI or pre-existing comorbidities.

Analyses of maternal age categories showed that the adjusted RRs increased with maternal age for most outcomes, except for SGA and PPH (**Table 3**). As compared with women younger than 35 years, the risks of PAS (RR = 1.60; 95% CI = 1.24-2.05), PP (RR = 1.80; 95% CI = 1.44-2.26), LGA (RR = 1.26; 95% CI = 1.09-1.47) and macrosomia (RR = 1.26; 95% CI = 1.09-1.45) were highest for women aged 40-44 years, and the risks of GDM (RR = 2.18; 95% CI = 1.70-2.80), HDP (RR = 3.04; 95% CI = 2.16-4.26), PE (RR = 2.96; 95% CI = 1.81-4.84), cesarean delivery (RR = 1.42; 95% CI = 1.19-1.70), PTB (RR = 1.70; 95% CI = 1.18-2.44) and congenital anomaly (RR = 2.86; 95% CI = 1.54-5.30) were highest for women aged 45 years or older. The sensitivity analysis showed similar results when missing values of covariates were imputed (data not shown).

**Table 3:** Associations of maternal age with maternal and neonatal outcomes*

	Maternal age, number % (95% CI)			
	**<35**	**35-39**	**40-44**	**≥45**
**Gestational diabetes mellitus**				
Unadjusted	Reference	1.52 (1.41-1.64)	1.86 (1.69-2.04)	2.43 (1.91-3.08)
Adjusted	Reference	1.48 (1.35-1.62)	1.77 (1.59-1.98)	2.18 (1.70-2.80)
**Hypertensive disorders of pregnancy**				
Unadjusted	Reference	1.81 (1.59-2.07)	2.15 (1.84-2.52)	4.45 (3.24-6.12)
Adjusted	Reference	1.49 (1.28-1.75)	1.68 (1.40-2.02)	3.04 (2.16-4.26)
**Preeclampsia**				
Unadjusted	Reference	1.75 (1.44-2.12)	2.16 (1.71-2.72)	4.53 (2.86-7.19)
Adjusted	Reference	1.43 (1.14-1.81)	1.65 (1.26-2.16)	2.96 (1.81-4.84)
**Placenta accreta spectrum**				
Unadjusted	Reference	2.99 (2.50-3.57)	3.81 (3.12-4.67)	5.28 (3.47-8.04)
Adjusted	Reference	1.40 (1.11-1.75)	1.60 (1.24-2.05)	1.50 (0.96-2.36)
**Placenta previa**				
Unadjusted	Reference	2.41 (2.04-2.83)	2.95 (2.44-3.58)	3.65 (2.34-5.69)
Adjusted	Reference	1.57 (1.28-1.91)	1.80 (1.44-2.26)	1.73 (1.09-2.76)
**Cesarean delivery**				
Unadjusted	Reference	1.50 (1.42-1.58)	1.79 (1.68-1.91)	2.04 (1.72-2.41)
Adjusted	Reference	1.21 (1.13-1.29)	1.39 (1.29-1.50)	1.42 (1.19-1.70)
**Postpartum hemorrhage**				
Unadjusted	Reference	1.49 (1.30-1.71)	1.63 (1.37-1.94)	1.97 (1.24-3.14)
Adjusted	Reference	1.15 (0.98-1.35)	1.13 (0.93-1.37)	1.28 (0.79-2.07)
**Preterm birth**				
Unadjusted	Reference	1.52 (1.35-1.71)	2.04 (1.77-2.35)	2.84 (2.02-4.00)
Adjusted	Reference	1.34 (1.16-1.55)	1.56 (1.32-1.86)	1.70 (1.18-2.44)
**Large for gestational age†**				
Unadjusted	Reference	1.14 (1.04-1.26)	1.37 (1.21-1.54)	1.26 (0.88-1.81)
Adjusted	Reference	1.14 (1.00-1.30)	1.26 (1.09-1.47)	1.06 (0.73-1.55)
**Small for gestational age†**				
Unadjusted	Reference	1.07 (0.91-1.26)	0.87 (0.70-1.09)	1.39 (0.79-2.43)
Adjusted	Reference	1.07 (0.87-1.32)	0.88 (0.67-1.15)	1.39 (0.77-2.50)
**Macrosomia**				
Unadjusted	Reference	1.36 (1.24-1.49)	1.61 (1.43-1.81)	1.53 (1.07-2.19)
Adjusted	Reference	1.13 (1.00-1.27)	1.26 (1.09-1.45)	1.06 (0.73-1.53)
**Congenital anomaly**				
Unadjusted	Reference	2.54 (1.97-3.27)	2.54 (1.87-3.44)	5.57 (3.14-9.88)
Adjusted	Reference	1.35 (0.99-1.85)	1.42 (0.99-2.04)	2.86 (1.54-5.30)

*Data are relative risk (95% confidence interval). Adjusted for ethnicity, education, occupation, annual household income, gestational age at enrolment, pre-pregnancy body mass index, smoking and alcohol consumption within six months prior to pregnancy, parity, method of conception, and preexisting comorbidities.

†Excluding missing data on birth weight, neonatal sex, and gestational age at delivery (n = 3750).

The associations between categorical maternal age and some outcomes varied by parity (Table S3 in the **Online Supplementary Document**) and COVID-19 (Table S4 in the **Online Supplementary Document**), but not by pre-pregnancy BMI and pre-existing comorbidities. The risks for HDP (*P* for interaction = 0.002), PE (*P* for interaction = 0.004), PP (*P* for interaction = 0.019), and cesarean delivery (*P* for interaction = 0.003) were more pronounced in nulliparous women than multiparous women, and the risks for GDM (*P* for interaction = 0.020) and PAS (*P* for interaction = 0.007) were more pronounced during COVID-19 than before COVID-19. For example, the increased risks of HDP (RR = 3.63; 95% CI = 1.24-10.68 vs RR = 2.45: 95% CI = 1.70-3.52), PE (RR = 7.43; 95% CI = 1.56-35.43 vs RR = 2.10; 95% 1.24-3.57), PP (RR = 4.42; 95% CI = 0.57-34.52 vs RR = 1.58; 95% CI = 0.97-2.56), and cesarean delivery (RR = 2.18; 95% CI = 1.14-4.18 vs RR = 1.31; 95% CI = 1.09-1.58) at age of ≥45 years were larger in nulliparous women than multiparous women.

## DISCUSSION

From this geographical diverse cohort of pregnant women in China, we found generally nonlinear and positive relationships between maternal age and risks of adverse pregnancy outcomes, with inflection points at 35.6-40.4 years. We also confirmed increased risks of adverse outcomes with increasing maternal age, which were more pronounced in nulliparous women than in multiparous women.

Consistent with previous studies [6,9,15,25,26], we found that older maternal age is independently associated with higher risks of adverse pregnancy outcomes, with highest risk observed in women of very advanced maternal age (40-44.9 years old) or extremely advanced maternal age (≥45 years old). Our work adds to previous studies by involving more outcomes, which allowed us to assess a wide range of maternal and perinatal outcomes, including GDM, HDP, PPH, PE, PAS, PP, cesarean delivery, PTB, LGA, SGA, macrosomia, and congenital anomaly. We also examined whether advanced maternal age is associated with increased risks among nulliparous women to the same extent as among multiparous women and found stronger associations in the former compared to the latter group, addressing a gap in which most previous studies were focused to nulliparous women [27-29], because of which an interaction with parity has not been reported previously. However, such an interaction needs to be replicated in other studies before we can conclude that maternal age really does have a different effect on adverse pregnancy outcomes in these two groups. Our findings highlight the importance of appropriate childbearing age for both maternal and offspring health, especially for women having their first child.

Our study also extends previous work on the relationships between maternal age continuum and adverse pregnancy outcomes. Although the shapes of the relationships differed, the risks of adverse outcomes increased with increasing maternal age, and inflection points were commonly observed at age 35.6 or age 40.4. For example, the inflection points for PAS, LGA, cesarean delivery, and PP were around the age of 36 years (range = 35.6-36.4), and the inflection points for GDM, PTB, macrosomia, HDP, PE, and congenital anomaly were around the age of 40 (range = 39.6-40.4). These findings support the current clinical and public health definitions that used age 35 as the threshold to determine advanced maternal age and age 40 as the threshold to determine very advanced maternal age.

Several different pathways, which are not mutually exclusive, could explain the association between maternal age and adverse outcomes. First, advanced maternal age was found to be associated with higher levels of insulin resistance, circulating adipokines, inflammatory markers, and oxidative stress [30], which could partly explain the association between maternal age and risk of GMD, HDP, and PE. Second, age-related weakening of the myometrium, reduction in the number of oxytocin receptors, increased rates of maternal systemic diseases and obstetric complications, and the lower clinical threshold for obstetric interventions could explain the higher risks of PPH and cesarean delivery in older women [31,32]. Third, advanced maternal age represents altered uterine, hormonal or implantation environment such as aging processes in the placental and myometrial vascular lesions and progesterone deficiency which were associated with PTB, PAS, and PPH [33,34]. Fourth, women with advanced maternal age may have higher pre-pregnancy BMI and excessive gestational weight gain, which may lead to LGA, macrosomia, and cesarean delivery. Finally, the mechanisms underlying the impact of maternal age on some adverse outcomes, such as PP, remain unclear and require further investigation. Although inconsistent results were reported for SGA in previous studies [9,35], we observed no significant association, possibly due to other factors such as nutritional or socioeconomic factors contributing to the risk of SGA.

One of the limitations of this study is our use of data from hospital-based cohort including only pregnant women from public referral hospitals, which would lead to biased effect estimates if these associations were differed from those in non-referral or private hospitals. Second, although we had detailed information about many potential confounders, residual confounding from other sociodemographic and lifestyle-related factors such as physical activity and dietary intake during pregnancy cannot be excluded. Third, because the UNIHOPE cohort was designed to study pregnancy outcomes in women with advanced maternal age, we were not able to examine association of very young maternal age (i.e. <20 years) with pregnancy outcomes. Births at this very young age are now not rare in China, although women over 35 years of age represent a larger and growing population group. Thus, a much greater focus on contraceptive availability and reproductive health education for adolescents is also warranted. Fourth, women with higher adherence to the antenatal examination were more likely to be included in the study, causing a possible selection bias with unknown direction and extent. Fifth, the data on smoking and alcohol consumption rely on self-reporting and are thus subject to biases and incorrectness (primarily under-reporting). Finally, we had limited statistical power to detect the differences in risks of some outcomes (such as PPH and SGA) across maternal age groups, especially for the ≥45 group.

## CONCLUSIONS

We found that older maternal ages are nonlinearly associated with greater risks of adverse pregnancy outcomes, with inflection points at around 35.6-40.4 years, supporting current definitions and thresholds of advanced (35 years) and very advanced maternal age (40 years). We also found that the risks associated with maternal age are more pronounced in nulliparous than multiparous women, indicating that parity needs to be considered when providing counselling on age of pregnancy. Future studies should examine the possible modification effects on these associations by maternal key characteristics such as parity, ethnicity, and pre-existing comorbidities.

## Additional material


Online Supplementary Document
