# Supplementary material for: Association of maternal age with adverse pregnancy outcomes: A prospective multicenter cohort study in China
Source: J Glob Health. 2023 Dec 1;13:04161. doi: 10.7189/jogh.13.04161 (PMC10691438; doi:10.7189/jogh.13.04161)
Supplement: Online Supplementary Document [file jogh-13-04161-s001.pdf]

Association of maternal age with adverse pregnancy outcomes: a prospective multicenter cohort study in China

Yubo Zhou, Shaohua Yin, Qing Sheng, Jing Yang, Jianmeng Liu, Hongtian Li, Pengbo Yuan, Yangyu Zhao

Appendix

Contents

Supplementary Tables 2

Supplementary Figures 8

Supplementary Tables

Supplementary table 1. Hospitals and participants involved in the study [*]

No.	Study hospitals [b]	Number of participants
1	West China Second University Hospital	3350 (18.1)
2	The Third Affiliated Hospital of Guangzhou Medical University	2868 (15.5)
3	Shengjing Hospital	2627 (14.2)
4	First Affiliated Hospital of Chongqing Medical University	2194 (11.9)
5	Tongji Hospital	2061 (11.1)
6	Obstetrics & Gynecology Hospital of Fudan University	1993 (10.8)
7	Peking University Third Hospital	1831 (9.9)
8	Peking University First Hospital	1571 (8.5)

* Data are presented as number (percentage) of deliveries unless otherwise indicated.

† Hospitals located in eastern region included Peking University Third Hospital, Peking University First Hospital, Obstetrics & Gynecology Hospital of Fudan University, and The Third Affiliated Hospital of Guangzhou Medical University. Hospitals located in central region included Shengjing Hospitals and Tongji Hospital. Hospitals located in western region included West China Second University Hospital, and First Affiliated Hospital of Chongqing Medical University.

Supplementary table 2. Knots for spline models by pregnancy outcomes

Knot Number	Percentiles	Maternal age (years)
**Postpartum hemorrhage/Placenta accreta spectrum/Placenta previa/Cesarean delivery/Preterm birth**		
1	10	29
2	50	36
3	90	40
**Gestational diabetes mellitus/Preeclampsia/Large for gestational age/Congenital anomaly**		
1	5	31
2	35	35
3	65	37
4	95	42
**Macrosomia**		
1	5	27
2	23	34
3	41	36
4	59	37
5	77	39
6	95	42
**Hypertensive disorders of pregnancy/Small for gestational age**		
1	2.5	26
2	18.33	32
3	34.17	35
4	50	36
5	65.83	37
6	81.67	39
7	97.5	43

Supplementary 3. Interactive effects of maternal age and parity on pregnancy outcomes *

Maternal age	Parity		*P* values for interaction
	Nullipara	Multipara	
**Gestational diabetes mellitus**			0.513
<35	Reference	Reference	
35-39	**1.52 (1.28-1.81)**	**1.45 (1.30-1.62)**	
40-44	**1.70 (1.30-2.22)**	**1.75 (1.55-1.99)**	
≥45	1.67 (0.60-4.67)	**2.21 (1.70-2.86)**	
**Hypertensive disorders of pregnancy**			**0.002**
<35	Reference	Reference	
35-39	**2.22 (1.67-2.95)**	1.19 (0.98-1.43)	
40-44	**3.06 (2.08-4.51)**	**1.30 (1.05-1.61)**	
≥45	**3.63 (1.24-10.68)**	**2.45 (1.70-3.52)**	
**Preeclampsia**			**0.004**
<35	Reference	Reference	
35-39	**3.00 (1.94-4.62)**	1.01 (0.78-1.33)	
40-44	**3.88 (2.14-7.03)**	1.15 (0.85-1.56)	
≥45	**7.43 (1.56-35.43)**	**2.10 (1.24-3.57)**	
**Placenta accreta spectrum**			0.595
<35	Reference	Reference	
35-39	**1.92 (1.13-3.27)**	**1.34 (1.04-1.73)**	
40-44	1.33 (0.51-3.52)	**1.56 (1.18-2.05)**	
≥45	3.15 (0.32-31.41)	1.41 (0.88-2.26)	
**Placenta previa**			**0.019**
<35	Reference	Reference	
35-39	**1.93 (1.22-3.07)**	**1.43 (1.15-1.77)**	
40-44	1.18 (0.53-2.63)	**1.69 (1.33-2.16)**	
≥45	4.42 (0.57-34.52)	1.58 (0.97-2.56)	
**Cesarean delivery**			**0.003**
<35	Reference	Reference	
35-39	**1.35 (1.19-1.54)**	**1.13 (1.05-1.22)**	
40-44	**1.87 (1.54-2.26)**	**1.29 (1.18-1.40)**	
≥45	**2.18 (1.14-4.18)**	**1.31 (1.09-1.58)**	
**Postpartum hemorrhage**			0.341
<35	Reference	Reference	
35-39	1.18 (0.90-1.54)	1.12 (0.92-1.37)	
40-44	0.95 (0.62-1.47)	1.15 (0.92-1.45)	
≥45	0.53 (0.07-4.01)	1.37 (0.82-2.27)	
**Preterm birth**			0.878
<35	Reference	Reference	
35-39	1.20 (0.89-1.62)	**1.38 (1.16-1.63)**	
40-44	1.28 (0.79-2.09)	**1.63 (1.34-1.97)**	
≥45	1.98 (0.47-8.36)	**1.72 (1.18-2.52)**	

**Large for gestational age** †			0.907
<35	Reference	Reference	
35-39	1.13 (0.83-1.55)	1.15 (0.99-1.33)	
40-44	1.34 (0.84-2.12)	1.26 (1.07-1.49)	
≥45	1.25 (0.28-5.61)	1.04 (0.7-1.54)	
**Small for gestational age** †			0.862
<35	Reference	Reference	
35-39	1.07 (0.74-1.55)	1.05 (0.81-1.37)	
40-44	0.81 (0.40-1.62)	0.88 (0.64-1.20)	
≥45	2.36 (0.52-10.79)	1.30 (0.68-2.48)	
**Macrosomia**			0.809
<35	Reference	Reference	
35-39	1.20 (0.92-1.57)	1.14 (0.99-1.30)	
40-44	1.35 (0.88-2.05)	**1.26 (1.08-1.47)**	
≥45	1.50 (0.35-6.41)	1.04 (0.71-1.53)	
**Congenital anomaly**			
<35	Reference	Reference	0.318
35-39	**2.31 (1.10-4.88)**	1.15 (0.82-1.63)	
40-44	2.65 (0.90-7.83)	1.23 (0.83-1.80)	
≥45	**15.46 (1.77-135.02)**	**2.41 (1.26-4.62)**	

* Data are relative risk (95% confidence interval). Adjusted for ethnicity, education, occupation, annual household income, gestational age at enrolment, pre–pregnancy body mass index, smoking and alcohol consumption within 6 months prior to pregnancy, parity, method of conception, and preexisting comorbidities.

† Data on missing information on birth weight, neonatal sex, and gestational age at delivery (n=3750).

Supplementary table 4. Interactive effects of maternal age and Covid-19 pandemic on pregnancy outcomes *

Maternal age	Covid-19 pandemic		*P* values for interaction
	Before	During	
**Gestational diabetes mellitus**			**0.020**
<35	Reference	Reference	
35-39	**1.42 (1.28-1.58)**	**1.55 (1.28-1.87)**	
40-44	**1.70 (1.51-1.93)**	**1.86 (1.42-2.44)**	
≥45	**2.07 (1.59-2.68)**	2.81 (0.92-8.57)	
**Hypertensive disorders of pregnancy**			0.323
<35	Reference	Reference	
35-39	**1.46 (1.22-1.75)**	**1.85 (1.33-2.57)**	
40-44	**1.68 (1.36-2.07)**	**1.87 (1.19-2.94)**	
≥45	**3.10 (2.18-4.40)**	NA	
**Preeclampsia**			0.636
<35	Reference	Reference	
35-39	**1.54 (1.17-2.02)**	**1.72 (1.07-2.74)**	
40-44	**1.80 (1.32-2.45)**	**2.01 (1.08-3.74)**	
≥45	**3.36 (2.01-5.61)**	NA	
**Placenta accreta spectrum**			**0.007**
<35	Reference	Reference	
35-39	1.22 (0.94-1.58)	**1.94 (1.20-3.13)**	
40-44	**1.42 (1.07-1.88)**	**2.03 (1.12-3.65)**	
≥45	1.35 (0.84-2.16)	1.33 (0.15-11.52)	
**Placenta previa**			0.260
<35	Reference	Reference	
35-39	**1.64 (1.29-2.07)**	1.12 (0.71-1.75)	
40-44	**1.83 (1.41-2.37)**	**1.91 (1.09-3.37)**	
≥45	**1.73 (1.06-2.83)**	2.83 (0.35-22.95)	
**Cesarean delivery**			0.575
<35	Reference	Reference	
35-39	**1.20 (1.12-1.29)**	1.14 (0.99-1.31)	
40-44	**1.37 (1.26-1.49)**	**1.38 (1.13-1.68)**	
≥45	**1.40 (1.17-1.69)**	1.44 (0.58-3.59)	
**Postpartum hemorrhage**			0.541
<35	Reference	Reference	
35-39	1.12 (0.94-1.33)	1.09 (0.69-1.72)	
40-44	1.08 (0.88-1.34)	1.37 (0.72-2.63)	
≥45	1.27 (0.78-2.07)	NA	
**Preterm birth**			0.540
<35	Reference	Reference	
35-39	**1.21 (1.02-1.44)**	**1.77 (1.32-2.37)**	
40-44	**1.43 (1.18-1.73)**	**1.98 (1.32-2.97)**	
≥45	**1.53 (1.05-2.24)**	2.38 (0.53-10.75)	

**Large for gestational age** †			0.488
<35	Reference	Reference	
35-39	1.12 (0.96-1.30)	1.19 (0.91-1.55)	
40-44	**1.22 (1.03-1.45)**	**1.43 (1.00-2.07)**	
≥45	1.06 (0.72-1.56)	NA	
**Small for gestational age** †			0.484
<35	Reference	Reference	
35-39	1.15 (0.91-1.45)	1.24 (0.76-2.02)	
40-44	0.99 (0.74-1.32)	0.61 (0.26-1.46)	
≥45	1.46 (0.78-2.70)	5.75 (0.69-48.03)	
**Macrosomia**			0.103
<35	Reference	Reference	
35-39	1.11 (0.97-1.28)	0.96 (0.75-1.23)	
40-44	**1.21 (1.03-1.42)**	1.32 (0.93-1.86)	
≥45	1.05 (0.72-1.54)	NA	
**Congenital anomaly**			0.192
<35	Reference	Reference	
35-39	1.04 (0.73-1.47)	1.99 (0.95-4.17)	
40-44	1.11 (0.75-1.65)	1.75 (0.62-4.94)	
≥45	**2.25 (1.19-4.25)**	NA	

* Data are relative risk (95% confidence interval). Adjusted for ethnicity, education, occupation, annual household income, gestational age at enrolment, pre–pregnancy body mass index, smoking and alcohol consumption within 6 months prior to pregnancy, parity, method of conception, and preexisting comorbidities.

† Data on missing information on birth weight, neonatal sex, and gestational age at delivery (n=3750).

Supplementary Figures

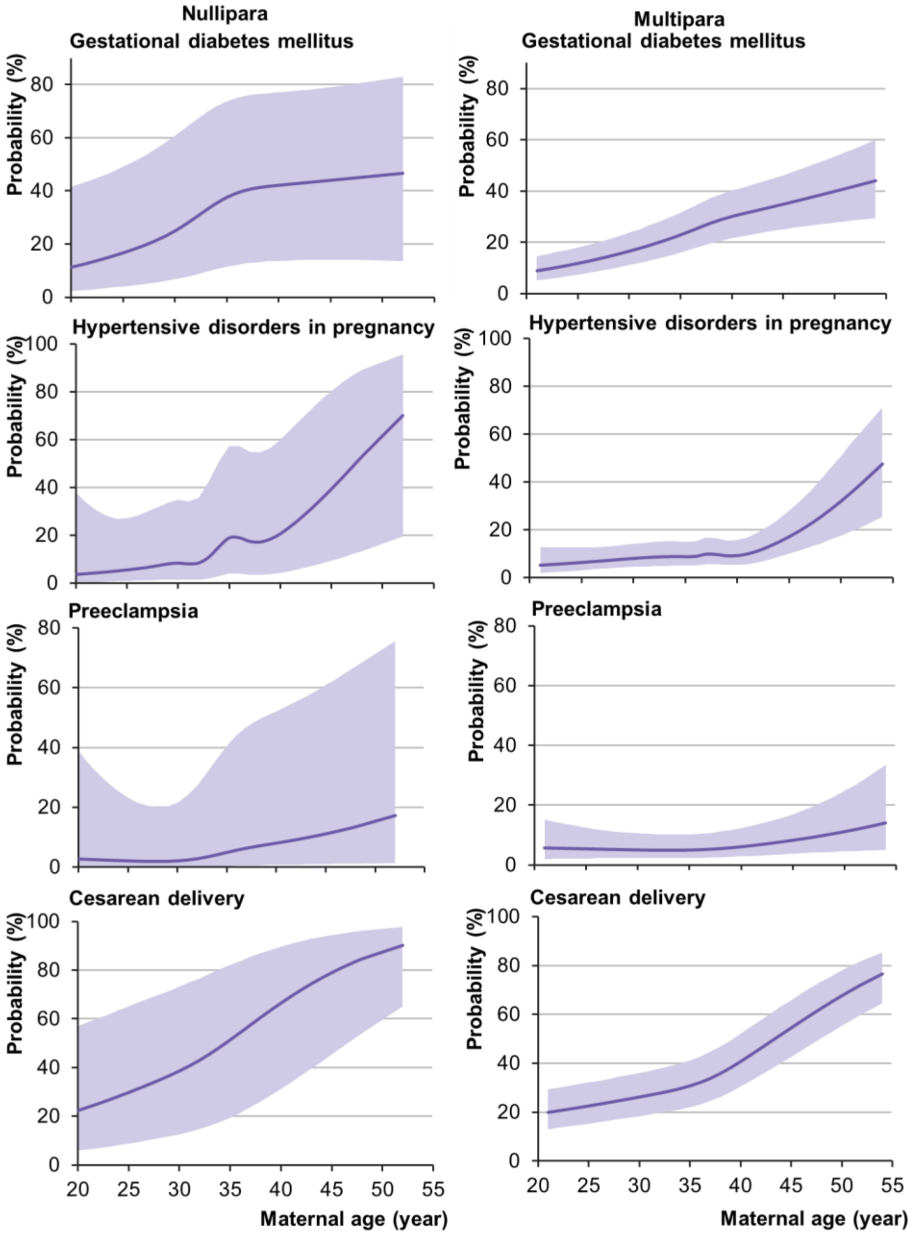

Supplementary figure 1. Predicted probabilities of adverse pregnancy outcomes across maternal age, stratified by parity. Predicted probabilities were transformed from the adjusted odds, which were calculated using multivariable logistic regression models, with adjustment for ethnicity, education, occupation, annual household income, gestational age at enrolment, pre-pregnancy body mass index, smoking and alcohol consumption within 6 months prior to pregnancy, method of conception, and preexisting comorbidities. Dark purple lines indicate predicted probabilities, and the purple bands represent 95% confidence intervals.

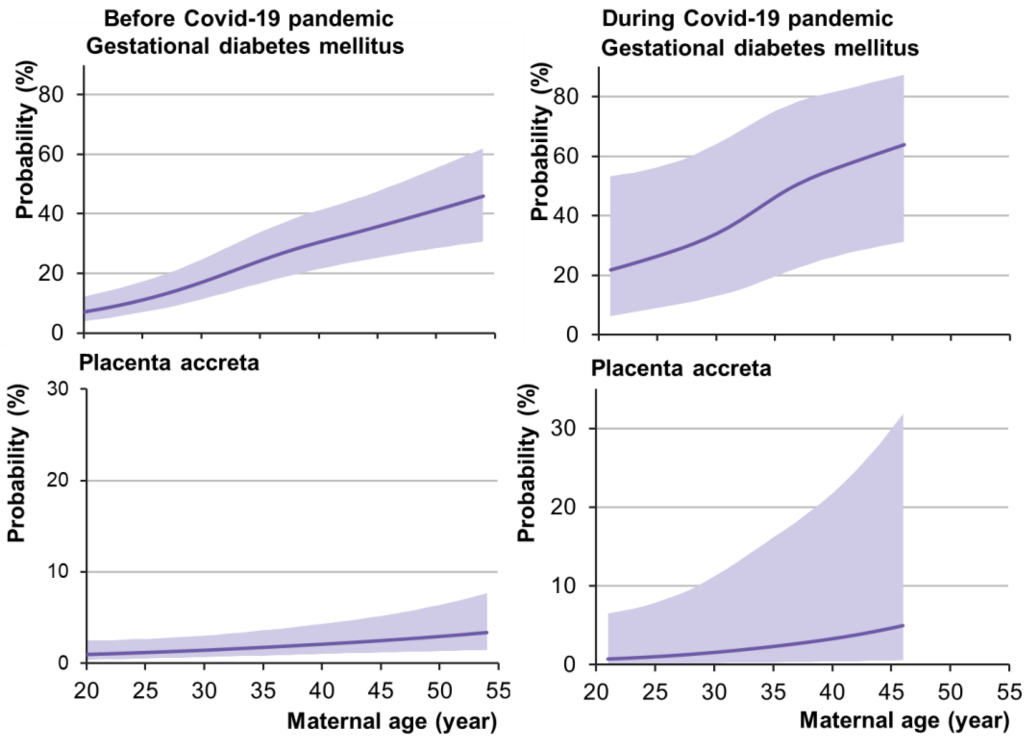

Supplementary figure 2. Predicted probabilities of adverse pregnancy outcomes across maternal age, stratified by Covid-19 pandemic. Predicted probabilities were transformed from the adjusted odds, which were calculated using multivariable logistic regression models, with adjustment for ethnicity, education, occupation, annual household income, gestational age at enrolment, pre-pregnancy body mass index, smoking and alcohol consumption within 6 months prior to pregnancy, parity, method of conception, and preexisting comorbidities. Dark purple lines indicate predicted probabilities, and the purple bands represent 95% confidence intervals.
